# TERIUS: accurate prediction of lncRNA via high-throughput sequencing data representing RNA-binding protein association

**DOI:** 10.1186/s12859-018-2013-9

**Published:** 2018-02-19

**Authors:** Seo-Won Choi, Jin-Wu Nam

**Affiliations:** 1 Department of Life Science, College of Natural Sciences, Hanyang University, Seoul, 04763 Republic of Korea; 2 Research Institute for Convergence of Basic Sciences, Hanyang University, Seoul, 04763 Republic of Korea

**Keywords:** LncRNA, LncRNA annotation, RNA binding protein association

## Abstract

**Background:**

LncRNAs are long regulatory non-coding RNAs, some of which are arguably predicted to have coding potential. Despite coding potential classifiers that utilize ribosome profiling data successfully detected actively translated regions, they are less sensitive to lncRNAs. Furthermore, lncRNA annotation can be susceptible to false positives obtained from 3′ untranslated region (UTR) fragments of mRNAs.

**Results:**

To lower these limitations in lncRNA annotation, we present a novel tool TERIUS that provides a two-step filtration process to distinguish between bona fide and false lncRNAs. The first step successfully separates lncRNAs from protein-coding genes showing enhanced sensitivity compared to other methods. To eliminate 3’UTR fragments, the second step takes advantage of the 3’UTR-specific association with regulator of nonsense transcripts 1 (UPF1), leading to refined lncRNA annotation. Importantly, TERIUS enabled the detection of misclassified transcripts in published lncRNA annotations.

**Conclusions:**

TERIUS is a robust method for lncRNA annotation, which provides an additional filtration step for 3’UTR fragments. TERIUS was able to successfully re-classify GENCODE and miTranscriptome lncRNA annotations. We believe that TERIUS can benefit construction of extensive and accurate non-coding transcriptome maps in many genomes.

**Electronic supplementary material:**

The online version of this article (10.1186/s12859-018-2013-9) contains supplementary material, which is available to authorized users.

## Background

Long non-coding RNAs (lncRNAs) are a group of regulatory non-coding RNAs (ncRNAs) that are involved in diverse biological processes [1]. Despite developments in research, lncRNA is still poorly defined and therefore suffers from erroneous annotation [2–4]. For one, the coding potential of lncRNA has long been debated, regardless of the fact that its name harbors the term “non-coding.” Several studies have reported unexplained associations between ribosomes and varying proportions of lncRNAs across different species and cell lines [5–7]. Meanwhile, other work has led to different conclusions, including that lncRNAs are deprived of functional open reading frames (ORFs) [8, 9] or that some lncRNAs are actively translated [6, 10, 11]. Other research reports that some lncRNAs are capable of coding short functional peptides in mice [12, 13], and that lncRNAs may be bifunctional, with coding and non-coding isoforms reacting to cell conditions [14, 15]. Unfortunately, the extent to which these ribosome-associated lncRNAs are translated remains unknown.

To resolve this question, pioneers in the lncRNA field developed several algorithms to identify translated transcripts by sensing their intrinsic characters, such as the ORF length [16–18], sequence similarity to known proteins [19], conservation [16, 20], and codon usage or kmer bias [16–18, 21]. More recently, following the footsteps of Ingolia et al. and Guttman et al. [9, 22], several studies have explored coding potential prediction within the context of in vivo translation using ribosome profiling data. Ingolia and his colleagues defined translation efficiency (TE) as the approximation of effective ribosome engagement to RNAs, and Guttman and his group conceived a program, the ribosome release score (RRS), focused on ribosome disengagement at the start of 3’UTR region. The translated ORF classifier (TOC) developed by Chew et al. also employed a similar feature [8]. Bazzini et al. devised the ORFscore that predicts the coding potential of ORFs by quantifying the biased distribution of ribosome reads toward the first frame by testing observed ribosome read distributions under the null hypothesis of Chi-squared test [23]. Subsequently, Calveilo et al. devised a more sophisticated program, RiboTaper, coupling ribosome periodicity with Fourier transformation strengthened by a multitaper approach [24]. Rather than imposing a hypothetical uniform distribution like in the ORFscore, RiboTaper compares the spectra derived from ribosome protected fragment sequencing (Ribo-seq) to those from high-throughput RNA sequencing (RNA-seq) to ensure capture of significant peaks of frequency representing periodicity. Despite that current strategies can be effective for the detection of conserved, highly expressed, classic protein-coding genes, they may not be appropriate for the identification of young, less productive genes with short ORFs that are the center of the ongoing debate.

Other than the ambiguity regarding the translated lncRNA subpopulation, the non-coding group also suffers from debatable annotations. As novel transcripts are assembled based on RNA-seq signals, where only the coding potential is assessed before they are annotated as lncRNAs, the integrity of lncRNA annotation is barely protected from the non-coding fragments of other genes. Above all, the 3’UTR region of protein-coding genes is the leading candidate for such fragments as it tends to show weak, long, and fragmented RNA-seq signals that stretch downstream. Nonetheless, no appropriate solution has been suggested for the detection of false annotations. Although the use of cap analysis of gene expression (CAGE) and polyadenylation tags for the determination of transcript boundaries has been quite effective [5, 25], the required data are not available for most model organisms and cell types.

To address these problems, we introduce the Translation-dependent Ensemble classifier with RIbosome and UPF1 association Score (TERIUS) that is robust and can successfully refine lncRNA annotations using a two-step paradigm. The first step, involves calculation of the ribosome periodicity score (RPS) and is responsible for separating coding transcripts. The second step, involves calculation of the UPF1 association score (UAS) that detects invalid lncRNAs.

## Methods

### Processing of high-throughput sequence data

All Ribo-seq and RNA-seq sequence data used in this study were downloaded from the NCBI gene expression omnibus (GEO) dataset [26] and aligned to reference genomes (hg19 for human and mm9 for mouse) using TopHat version 2.0.6 [27] with alignment options -g 1 --b2-N 0 --b2-L 20, intron options -i 61 -I 265006 for the human sequence, and -i 52 -I 240764 for the mouse sequence (Additional file 1: Table S1).

Crosslinking immunoprecipitation sequencing (CLIP-seq) BED files were downloaded from the GEO dataset, converted to BAM files using BEDtools version 2.17.0 [28], and then modified with an in-house Python script to add reads according to BED signal intensity. Three mouse CLIP-seq replicates were merged and the mean values were obtained. All gene expression or association levels were calculated using an in-house Python script.

CAGE-seq and poly(A)-position profiling by sequencing (3P–seq) BED files were downloaded from the FANTOM 5 project [29] and from NCBI GEO datasets (Additional file 1: Table S1).

### Training and test datasets

To define the training and test gene annotations for TERIUS, the RefSeq protein-coding gene annotation (version 2013.09.09 for human and 2014.11.23 for mouse) [30], GENCODE annotation version v19 [2] for ncRNA genes, and Vertebrate Genome Annotation (VEGA) lncRNA annotation (version 54 for human and version 68 for mouse) [31] were downloaded. Annotations based on mm10 genome assembly were converted to the mm9 assembly using the liftOver tool [32]. Collected annotations were then subjected to downstream processes for RPS followed by UAS.

For the RPS, the protein-coding gene isoforms with the longest coding sequence (CDS) were selected. To generate positive (non-coding) data, classical non-coding RNAs (rRNA, snoRNA, snRNA, miRNA, and so on) were collected from GENCODE v19 annotations. Among collected ncRNA annotation, those with protein-coding potential was filtered out using CPC [19] and CPAT [18]. Default cutoffs and logistic model provided by each program were used. Remaining ncRNAs were again searched against UniProt database [33] to eliminate any genes with reported protein-coding entries. Then for each coding and non-coding gene, Ribo-seq signals for the sub-codon positions were counted and those that lacked signal in any of the three positions were discarded, leaving 7710 mRNAs and 92 ncRNAs. To account for the size imbalance between ncRNAs and protein-coding genes, the same number of protein-coding genes as ncRNAs (92) were randomly sampled to generate 50 replicate training cohorts with the same training set of ncRNAs.

For the UAS, classical non-coding RNAs were downloaded from ENSEMBL versions 75 and 67 for mouse genome [34]. LncRNA annotations from VEGA database were compared with RefSeq protein-coding transcripts and ENSEMBL ncRNAs to remove lncRNAs that overlap exons of any other genes in the same strand. Filtered lncRNA transcripts were selected for the longest isoform to generate positive set. For negative set, the isoforms of protein-coding genes with 3’UTR regions that did not overlap with the CDS of any other isoforms were selected. If more than one isoform remained, the one with the longest 3’UTR region was selected. Both the lncRNA and 3’UTR region of the mRNA were required to be expressed more than or equal to RNA-seq RPM of 0.3. To control the length and size difference between the 3’UTR set and the lncRNA set, the 3’UTR region was randomly fragmented into the same length as the lncRNAs in a pairwise manner. The process was then repeated 10 times to generate replicates with lncRNAs and length- and number-matched 3’UTR fragments. The resulting dataset consisted of 1334 3’UTR fragments and 1334 lncRNAs from the human genome and 290 3’UTR fragments and 290 lncRNAs from the mouse genome.

To assess the overall performance of TERIUS, lncRNA annotations from the miTranscriptome, BIGTranscriptome, and GENCODE versions 19 and M1 were downloaded and the longest isoforms were selected [2–4]. Genes without strand information in miTranscriptome were excluded from downstream analysis.

### Prediction of the most-likely reading frame

**Fig. 1:**
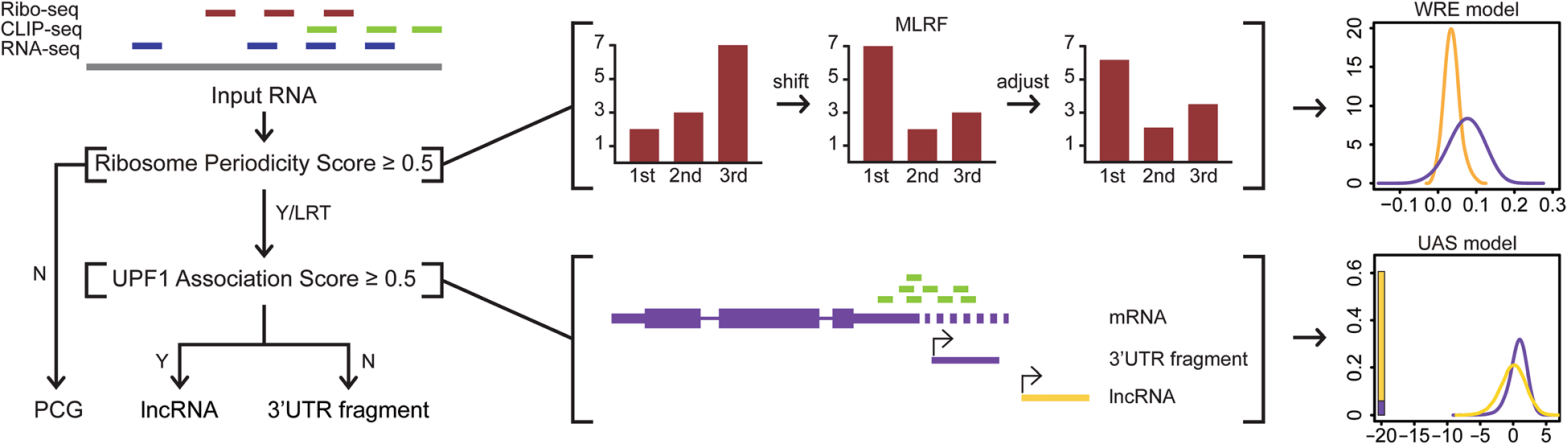
Two-step schematic flow of TERIUS. In RPS, ribosome reads are mapped to transcripts and converted to sub-codon position signals. Then the signals are shifted to find the most-likely coding frame and adjusted before weighted relative entropy was calculated for ncRNA (gold) and mRNA (purple) sets. Resulting distribution was estimated to generate a model (x axis: WRE, y axis: density). Transcripts predicted as coding are classified as mRNA while ncRNA and low ribosome transcripts (LRT) are passed on to the second step, where they are further classified as bona fide lncRNAs or 3’UTR fragments depending on their association to UPF1. UAS classification is also based on density model. X axis represents UPF1 CLIP-seq RPM divided by RNA-seq RPM in log scale and y axis is density. The bar colored in yellow and purple in the left represents the fraction of transcripts without UPF1 association

To select the most-likely reading frame (MLRF) of transcripts, Ribo-seq reads with the same 5′-end positions were collapsed and counted by the sub-codon position in the 0-frame relative to the 5’end of transcript. To compare the obtained signals to the ones observed from the CDS regions of protein-coding genes, a weighted form of relative entropy (WRE) was designed as follows:

$$ WRE(f)=\sum \limits_{i=0,1,2}{obs}_f(i){ RIB O}_{CDS}(i)\mathit{\log}\frac{RIB{O}_{CDS}(i)}{RN{A}_{CDS}(i)} $$

where *obs*_*f*_(*i*) indicates the signal observed from the input transcript from sub-codon position *i* of frame *f*, and RIBO_CDS_(*i*) and RNA_CDS_(*i*) (i.e., the signal in the corresponding sequence data (Ribo-seq and RNA-seq respectively) obtained from the sub-codon position *i* over CDS of all protein-coding genes). The *WRE* was calculated for all three frames and the one with the highest value was regarded as the *MLRF* in downstream analysis (Fig. 1).

$$ MLRF=\underset{f\in \left\{0,1,2\right\}}{\mathrm{argmax}} WRE(f) $$

### Ribosome periodicity score (RPS)

The first step of TERIUS considers trinucleotide periodicity of ribosomal occupancy, a well-characterized signal of active translation. Relative entropy was applied to measure the periodicity by means of uneven ribosome signal intensity that favors the first sub-codon position over others. Instead of searching for all possible ORFs in the transcripts, TERIUS defines a “*MLRF*” by calculating ribosome signals over sub-codon positions for three possible frames. As this approach is prone to generate false signal enrichment in the first sub-codon position, additional normalization should be carried out before calculating the *WRE* value after the *MLRF* was decided. For all input transcripts, counted Ribo-seq reads were randomly re-distributed to three positions, and the bias of the Ribo-seq reads was measured as the random controls (Additional file 2: Figure S1a). Then, the observed signal was divided by the random control signal as shown below:

$$ \mathit{\operatorname{norm}}(i)=\frac{obs(i)}{\mathit{\operatorname{rand}}(i)}/\sum \limits_i\frac{obs(i)}{\mathit{\operatorname{rand}}(i)} $$

where *norm(i)* and *rand(i)*, each stands for normalized signal and randomly generated signal for each sub-codon position *i*. Normalized signals of RNA-seq showed uniform distribution, which indicates that the normalization was successful. Therefore, the normalized signals were used to calculate the *WRE* for ncRNAs and mRNAs and their density was estimated to build a representative model (Fig. 1; Additional file 2: Figure S1a). Finally, the translation status of the transcripts was inferred in the form of posterior probability (*RPS*) as follows:

$$ RPS=P\left( nc|\theta \right)=\frac{P\left(\theta | nc\right)P(nc)}{P\left(\theta | nc\right)P(nc)+P\left(\theta | PCG\right)P(PCG)} $$

where *θ* refers to the *WRE* value of a transcript and nc stands for ncRNA and PCG stands for protein-coding genes. The prior probability of the ncRNA and protein-coding genes was set to 0.5 equally, and the likelihood was calculated from the estimated density model (Fig. 1).

### UPF1 association score (UAS)

The second step of TERIUS utilizes another RNA-binding protein, UPF1 that discriminates between non-coding 3’UTR fragments and lncRNAs due to the translation-dependent translocation of UPF1 to the 3’UTR [35, 36]. To simulate the biological and physical properties of the 3’UTR fragments in assembled transcriptomes, all 3’UTR regions of the protein-coding genes were randomly fragmented. Following random fragmentation, the intensity of the UPF1 association was investigated by means of CLIP-seq RPM normalized by expression levels represented by RNA-seq RPM. Because of the major difference between the number of UPF1-present and -absent transcripts in the 3’UTR fragment population and the lncRNA population, this ratio was also considered as a part of the density model (Fig. 1; Additional file 2: Figure S1b). Thus, the posterior probability (*UAS*) was calculated as shown below:

$$ UAS=P\left( lnc|\theta \right)=\frac{P\left(\theta | lnc\right)P(lnc)}{P\left(\theta | lnc\right)P(lnc)+P\left(\theta | UTR\right)P(UTR)} $$

where lnc and UTR represent lncRNA and 3’UTR fragments respectively. As mentioned above, when there was no UPF1 association detected for a given transcript, the frequency of such transcripts in the lncRNA pool was used instead of the likelihood. The prior probability of the lncRNA and 3’UTR was set to 0.5.

### Model building and cross-validation

Once the *WRE* was calculated for the ncRNA and mRNA sets, their distribution was evaluated using the kernel density estimation function in R. The estimation function was trained by 5 × 2 nested cross-validation and the mean distribution of 50 replicate training datasets was used as a final model for *RPS*. Similarly, density distributions of *UAS* from lncRNA and mRNA were estimated and validated using a 5 × 2 nested cross-validation and the mean distribution of 10 replicate sets was used. Validation of the model was achieved using the outer fold of the nested cross-validation. Hyperparameters such as kernel function, bandwidth, and bandwidth adjustment were optimized for both classifiers using inner fold and are explained in Additional file (Additional file 1: Table S2).

### Benchmarking of alternative methods

RPS was mostly compared with ORFscore and RiboTaper as they share a key characteristic, which is trinucleotide periodicity. Performances of RPS and RiboTaper throughout this paper is based on default cutoff (0.5 for RPS and 0.05 for RiboTaper). For ORFscore, a heuristic cutoff was applied as the authors of ORFscore recommended. Scores were calculated for all RefSeq protein-coding genes using R code provided by the author [37] and the 15th percentile score was applied as in the original paper.

RiboTaper was downloaded from the website provided by the authors [24]. For RiboTaper, annotations files were created without CCDS (consensus CDS) and Appris tag. As RiboTaper requires selection of read lengths and P-site offsets for ribosome protected reads, those with lengths 23, 26, 29, 31, and 32 nt were used and all offsets were set to 12 nt.

## Results

### Current issues with lncRNA annotation

We first sought to ascertain the existence of a ribosome-associated lncRNA subpopulation. Consistent with previous reports, the analysis of ribosome profiling data revealed that a small portion of manually curated VEGA lncRNAs were associated with ribosomes in both humans and mice (Additional file 2: Figure S2a).

Next, the extent to which lncRNAs could potentially be 3’UTR fragments was assessed in GENCODE version 19 and version M1. Surprisingly, several thousands of lncRNAs were located within 100 kb downstream of the 3’UTR of GENCODE mRNAs in the same strand (Additional file 2: Figure S2b-c). A lncRNA AC006547.8 even overlaps 10 bp of the end of TRMT2A 3’UTR. Among those that were located near 3’UTR ends, more than half of the transcripts did not have CAGE tag evidence of transcription start site (Additional file 2: Figure S2c). It is therefore highly likely that the 3’UTR fragment could be a main source of non-coding contaminant in lncRNA annotations, especially as previous classifiers are only designed to detect the ability of a transcript to be translated and cannot discriminate between lncRNAs and parts of the coding transcripts, including 3’UTR regions. (Additional file 2: Figure S2d).

### Development of TERIUS classifier

To address two separate issues discussed above, we designed a program with two-step paradigm that resolves each issue per step. TERIUS consists of RPS, which detects actively translated transcripts, and UAS, which separates possible 3’UTR fragments from lncRNAs (Fig. 1). Protein coding potential is assessed per input transcripts using ribosome profiling data. After RPS step, the transcripts that are either predicted to be non-coding by RPS or that are associated insufficiently with ribosome are considered to be non-coding and passed on to UAS step. UAS computes UPF1 association based on UPF1 CLIP-seq data and RNA-seq data and predicts 3’UTR fragments and lncRNAs. The classification is based on the density model of each RPS and UAS scores that are developed using training dataset (Fig. 1).

### Performance of RPS classifier

**Fig. 2:**
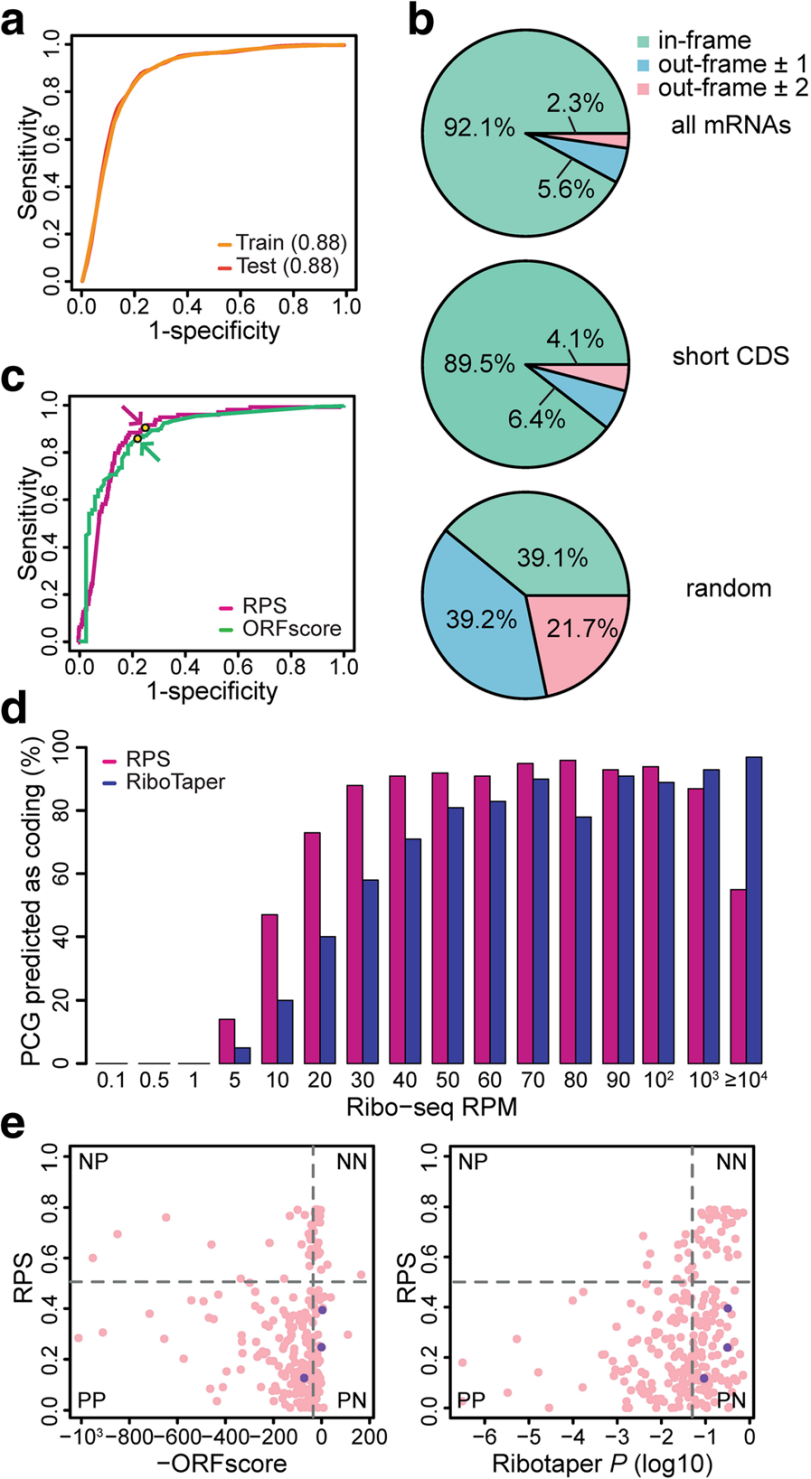
Performance of the RPS classifier (**a**) Training and test ROC curves of the RPS. (**b**) Accuracy of the most-likely reading frame (MLRF). The difference between the CDS frame and MLRF for all human mRNAs (top) and those harboring a short CDS are shown separately (middle). The prediction using RNA-seq is shown at the bottom (random). (**c**) Benchmarking ORFscore. AUC values are 0.88 for both RPS and ORFscore. Dots and arrows indicate the cutoff used to assess performance throughout the paper. (**d**) Percent of mRNAs predicted as coding by the RPS and RiboTaper. The mRNAs were binned according to Ribo-seq RPM. (**e**) 2D plot of the RPS versus the ORFscore (left) and RiboTaper (right). Dotted grey lines are the RPS cutoff (0.5), RiboTaper cutoff (0.05), and 15th percentile of the ORFscore, calculated using all RefSeq protein-coding genes. 11 outliers (ORFscore > 1000) are excluded from the plot. NP written at the top-left corner indicates genes plotted in the quadrant are classified as ncRNA (N) by RPS and as protein-coding (P) by other methods, as plotted along the x-axis

With the established density models (Fig. 1; Additional file 2: Figure S1b), we proceeded to investigate the performance of RPS and UAS in more detail. RPS showed robust performance in terms of AUC in both train and test sessions (Fig. 2a). To examine the accuracy of RPS and its strategy to define MLRF, the difference between the annotated CDS frame and the RPS-predicted frame was scrutinized for RefSeq protein-coding genes. The results showed that RPS correctly predicted reading frames for 92.1% of the protein-coding genes (Fig. 2b). For the remaining 5.6% and 2.3% of protein-coding genes, RPS defined other frames that were shifted 1 nt or 2 nt from the annotated CDSs due to low ribosome signals (Additional file 2: Figure S3a). Apart from low ribosome signals, it was already widely known that CDSs with short length tend to pass through cutoffs and are undetected during coding potential assessment. Therefore, among the RefSeq protein-coding genes that were analyzed, 218 genes with CDSs shorter than 100 aa were selected and separately analyzed. The results showed that RPS predicted the correct coding frames for 89.5% of the genes harboring short CDSs (Fig. 2b). To serve as a negative control and demonstrate that coding frame prediction power is truly based on ribosome signal periodicity, a similar analysis was carried out with RNA-seq reads. As expected, the coding frames RPS predicted using RNA-seq reads were randomly distributed throughout all three possible frames (Fig. 2b).

Next, to compare our results with previously published methods, all protein-coding and ncRNAs with ribosome signals in all sub-codon positions were subjected to ORFscore and RiboTaper, which are also built on the concept of ribosome read periodicity (Fig. 2c). ORFscore showed similar results as RPS with the same AUC values (0.88). Notably, RiboTaper failed to give results for 51.7% of the protein-coding genes (3982 out of 7710) and 85.9% of the ncRNAs (79 out of 92). As RiboTaper provided insufficient number of negative data (13 ncRNAs), RiboTaper was excluded in the ROC analysis. To further examine this low sensitivity issue, RefSeq protein-coding genes with at least one Ribo-seq read (Ribo-seq RPM>0) were divided into bins according to their RIBO-RPM (Fig. 2d). Results revealed that the RiboTaper missed more than half of the protein-coding genes with relatively low ribosome reads (RIBO-RPM ≤ 20) due to its rigorous filtering process upon annotation and sequence data.

### Validation of RPS classifier

Along with protein-coding gene classification, lncRNA classification was taken into account. To gather genes that were previously annotated as lncRNA but were recently found to have protein-coding evidence, genes with a symbol as ‘evidence at protein level’ were downloaded from Swiss-prot and GENCODE annotations. Resulting set had 124,764 human transcripts. To ensure correct comparison, we separated 1445 human genes with a single isoform and compared the performance of RPS to others in a pairwise manner (Fig. 2e). Out of 1445 genes, NCBP2-AS2, TDPX2 and TM4SF2 were not annotated as protein-coding in RefSeq. RPS and ORFscore each predicted three and two genes as protein-coding, but RiboTaper predicted all three genes as non-coding (Fig. 2e, purple dots). Although the additional contribution of RPS may seem small compared to ORFscore and RiboTaper, analysis of GENCODE ncRNA transcripts processed by each program suggests that those three methods address quite different sets of transcripts (Additional file 2: Figure S3b). Only 8 out of 92 human ncRNA were processed by all three methods (8.7%). Therefore, RPS still captures considerable population of ncRNAs that other methods cannot.

### Integrity of UAS classifier

**Fig. 3:**
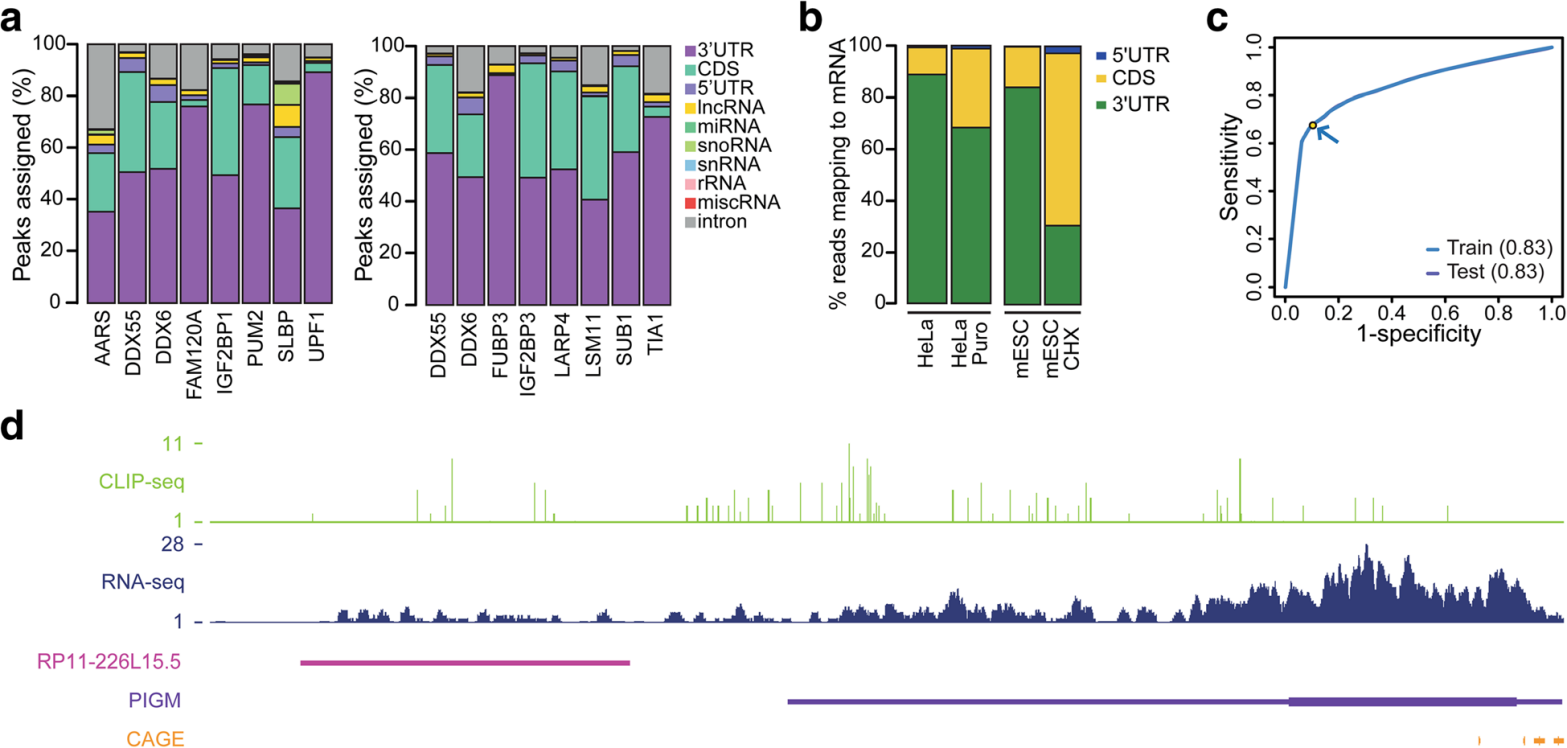
Utilizing RBPs showing translation-dependent association. (**a**) Association profiles of proteins that bind preferentially to 3’UTR of protein-coding genes in K562 (left) or HepG2 (right) cell line. (**b**) Translation-dependent localization of UPF1 to 3’UTR. Data are shown for untreated and puromycin (puro) or cycloheximide (CHX) treated HeLa cell (left) and mouse embryonic stem cell (mESC). (**c**) Train and test ROC curve of UAS. Dot and arrow indicate the cutoff point (0.5) used in this study. (**d**) Example of 3’UTR fragment annotated as lncRNA in GENCODE version 19. Data tracks below are CLIP-seq, RNA-seq and CAGE signal

While RPS relies on previously reported ribosome periodicity, the concept of using RNA-binding protein other than the ribosome is completely novel in terms of the assessment of biological coding potential. To identify those RNA-binding proteins that differentially associate with coding transcripts, recently published eCLIP dataset from the ENCODE project website [38] was used in BED format and low-quality peaks (*P* > 10^− 5^) were filtered out to profile the overall association of proteins to various gene types in GENCODE annotation. The result yielded clusters of proteins preferentially associated with 5’UTRs, CDSs, 3’UTRs, lncRNAs, miscRNAs, snoRNAs, and introns (Additional file 2: Figure S4a-f). Among them, a total of 14 RNA-binding proteins appeared to preferentially interact with the 3’UTR of protein-coding genes in K562 and HepG2 cell lines, including UPF1 and TIA1 (Fig. 3a) that are well-known for their characteristic preference for 3’UTR binding from previous studies [35, 36, 39]. Moreover, UPF1 is known to be the most highly enriched in mRNA 3’UTRs and is depleted in lncRNAs [35]. These findings were corroborated as UPF1 consistently demonstrated 3’UTR-enrichment and lncRNA-depletion in the annotation sets used in this study (Additional file 2: Figure S4g). Mouse embryonic stem cell (mESC) CLIP-seq data showed an increased proportion of introns, which may originate from noise. In lncRNA genes, both the HeLa and mESC profiles showed slightly more UPF1 abundance than eCLIP profiles although this was restricted to a small number of lncRNAs (Additional file 2: Figure S4 h), some of which were reported to interact with UPF1 in a previous study [35]. Interestingly, UPF1 was reported to show translation-dependent translocation from the CDS to the 3’UTR in two studies [35, 36]. Analyzing the UPF1 CLIP-seq data confirmed that UPF1 indeed showed translation-dependent localization to the 3’UTR in HeLa and mESC cell lines (Fig. 3b), forgoing the conclusion that UPF1 is a valid RNA binding protein candidate that can separate mRNA 3’UTRs from ncRNAs.

Following model development, the overall performance of UAS was measured using ROC analysis. UAS revealed unbiased results with an AUC of 0.83 for both the training and the test set, while the AUC in the mouse model was 0.78 for training ROC and 0.79 for test ROC (Fig. 3c; Additional file 2: Figure S5a). As there is no currently available program to benchmark, we could not compare our UAS with others. Instead, to verify that UAS can indeed detect 3’UTR fragments, we applied UAS classifier to the lncRNA annotation from GENCODE version 19. UAS predicted 423 out of 3057 transcripts (13.84%) as 3’UTR fragments. An example of one of the lncRNA transcripts with the lowest posterior probability *RP11-226 L15.5* (*ENST00000562313.1*) is located 878 bp downstream of the *PIGM* 3’UTR without CAGE tag (Fig. 3d). CLIP-seq and RNA-seq signals continuously covers the whole *PIGM* gene and the lncRNA *RP11-226 L15.5,* implying that the lncRNA might be 3’UTR fragment of *PIGM* gene*.* Similar examples were observed in GENCODE version M1 lncRNA (Additional file 2: Figure S5b).

### TERIUS enhances lncRNA annotation

**Fig. 4:**
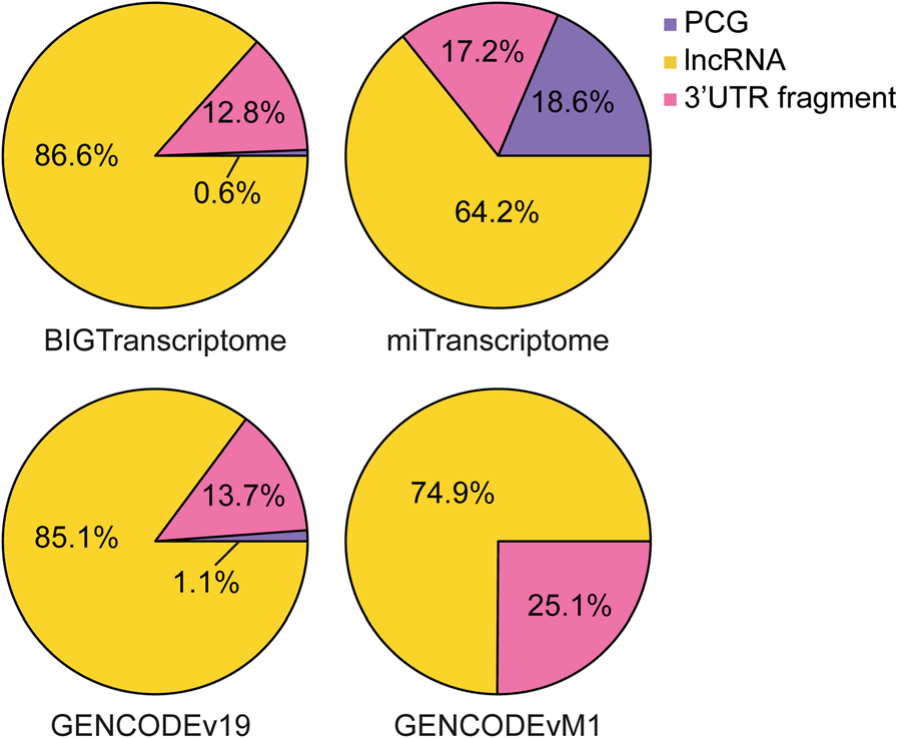
TERIUS can refine current lncRNA annotations. Re-classification results of miTranscriptome, BIGTranscriptome and GENCODE v19 lncRNAs and UAS classification of GENCODE vM1 lncRNAs are shown. PCG stands for protein-coding genes

As both RPS and UAS had proven to be fully capable of classifying lncRNA, the robustness of the ensemble form of the two algorithms was assessed. Using TERIUS, we reclassified lncRNAs originally defined by GENCODE v19, the miTranscriptome, and the BIGTranscriptome, the latter serving as the gold standard to estimate the accuracy of TERIUS. Of the miTranscriptome annotations, 77,014 with strand information were used for the downstream analysis. Comparing the results for these four lncRNA sets revealed that TERIUS identified 86.6% of the BIGTranscriptome lncRNAs correctly, indicative of powerful detection of bona fide lncRNAs (Fig. 4). On the contrary, only 64.2% of the miTranscriptome lncRNAs were classified as lncRNAs, indicating that the miTranscriptome contained a larger population of false lncRNAs than the BIGTranscriptome. Strikingly, RPS predicted 18.6% of miTranscriptome genes as protein-coding while the corresponding proportion in the BIGTranscriptome was only 0.6%. UAS classification results also highlights the difference between BIGTranscriptome and miTranscriptome, where 12.8% and 17.2% of lncRNAs, respectively, were predicted as 3’UTR fragments. The relatively higher portion of mRNA and lower portion of 3’UTR fragments of miTranscriptome may be the consequence of a previously reported tendency of the miTranscriptome, where nearby transcripts are often mistakenly fused into one gene model [4]. To ensure that UAS can detect fragmented transcripts, lncRNAs annotated in GENCODE vM1 was tested with UAS (Fig. 4). UAS discovered 25.1% of GENCODE vM1 lncRNAs as 3’UTR fragments, suggesting that a proportion of the non-coding contaminants in the miTranscriptome did not result from spurious classification of UAS. From inspection of miTranscriptome lncRNAs that are classified as 3’UTR fragments, those lncRNA without CAGE tag support or exon junction support were considered aberrant (Additional file 2: Figure S6). Furthermore, both RPS and UAS predicted that the ratio of protein-coding and 3’UTR fragments in the GENOCDE v19 annotation was compatible to that of the BIGTranscriptome, thus proving that TERIUS can enhance miTranscriptome lncRNA annotation.

## Discussion

Assembly and annotation of lncRNAs has become the general practice for discovery of novel key regulatory RNAs in various species. To understand lncRNA function, high-quality annotation and accurate coding potential assessment are crucial. TERIUS can benefit those studies attempting to widen the lncRNA reservoir by facilitating the process of lncRNA classification. TERIUS efficiently predicts non-coding transcripts including ones that are either missed out or predicted incorrectly by existing methods. TERIUS can also support lncRNAs that lack CAGE tags due to their low expression. The greatest strength of UAS score is that data of any other protein that shows similar affinity profile as that of UPF1 can be directly applied to UAS. In the Results section, proteins such as TIA1 are suggested as one of the possible alternatives. Nevertheless, there is an ever-growing need for more precise and specific definition of lncRNAs in order to better characterize and understand their biological properties. Even though the success of TERIUS suggests UAS as new criteria for lncRNA annotation, further research is required to expand annotation methods to allow for extensive identification of lncRNAs.

## Conclusions

In this study, we propose TERIUS as a robust and novel tool to eliminate false lncRNA annotations. We demonstrated that TERIUS can efficiently identify protein-coding transcripts and detect possible 3’UTR fragments within several public lncRNA annotations.

## Availability and requirements

TERIUS is publicly available with a Creative Commons Attribution-NonCommercial-ShareAlike 4.0 international license from //big.hanyang.ac.kr/TERIUS and is also included within the article [Additional file 3]. TERIUS is implemented in Python, runs on Linux CentOS 6.5 version. TERIUS requires Python (version 2.6.9), R (version 2.15.1) and Samtools (version 0.1.19-44,428 cd). TERIUS also requires Numpy (version 1.9.2) and Rpy (version 1.0.3) Python packages.

## Additional files

Additional file 1: Table S1. Accession numbers of NCBI GEO data sets used in this study. **Table S2**. Hyperparameters (kernel, bandwidth, adjustment) of kernel density estimation (PDF 225 kb)

Additional file 2: Figure S1. Building RPS and UAS model. (a) Correcting noise in sub-codon position signals. Raw, random and normalized signals of protein-coding genes are colored in purple, and those of ncRNAs are colored in gold. (b) Estimated association density model of lncRNA (gold) and 3’UTR fragments (purple) using mouse data. **Figure S2.** LncRNAs can be protein-coding or fragments of 3’UTR. (a) Proportion of human protein-coding genes (left) and VEGA lncRNAs (right) associated with Ribo-seq reads (top). Shown in the bottom are same results of mouse genes. (b) Distance between lncRNA start and 3’UTR end of protein-coding genes within 100 kb upstream of lncRNAs (left: human, right: mouse). Frequency of lncRNAs located within 100 kb downstream of 3’UTR are colored in red. (c) Percent of GENCODE v19 lncRNAs (top) located 100 kb downstream of 3’UTR of sense protein-coding gene (purple). Among them, those with CAGE tag supporting their 5’end is shown in blue. Below are the corresponding results of GENCODE vM1 lncRNAs. (d) Classification of 3’UTR regions using ORFscore and RiboTaper. The number on the top of each bars and the portion colored in yellow indicate the number of 3’UTR regions predicted as coding by each method. **Figure S3.** Performance of RPS compared to ORFscore and RiboTaper. (a) Ribosome read signals of RefSeq protein-coding genes, binned according to the difference between its CDS frame and its predicted frame. (b) Venn diagram depicting GENCODE v19 ncRNA subsets detected by RPS, ORFscore and RiboTaper. **Figure S4.** Association profiles of RBPs with eCLIP dataset and UPF1 CLIP-seq. Proteins that mostly bind to 5’UTR (a), CDS (b), lncRNA (c), miscRNA (d), snoRNA (e) and intron (f) are shown for eCLIP data of K562 and HepG2 cell lines. snoRNA and miscRNA-dominant proteins are shown for K562 cell lines only. (g) Detailed association profile of UPF1 to various genes in HeLa (left) and mESC (right) cell lines. Flanking 5 and Flanking 3 refers to the region 3 kb outside 5’UTR or 3’UTR. (h) Top 5 lncRNAs with UPF1 association in HeLa (left) and mESC (right) cell lines. LncRNAs with previously reported UPF1 association are colored in red. **Figure S5**. Development of UAS with mESC data. (a) Train and test ROC curves of mouse UAS model. Dot and arrow indicate the cutoff point (0.5) used in this study. (b) Example of false GENCODE vM1 lncRNA detected by UAS. Data tracks below are CLIP-seq and RNA-seq. No CAGE signal was visible within the region. **Figure S6.** Example of false lncRNA annotation in miTranscriptome detected by TERIUS. Example of miTranscriptome lncRNAs (magenta) that are possible 3’UTR fragments. 3’UTR end of RefSeq mRNA MEMO1 is shown in purple. There were no CAGE-seq tag visible in the region. (PDF 2195 kb)

Additional file 3: TERIUS source code. (GZ 19356 kb)

## Abbreviations

3’UTR: 3′ untranslated region 3P–seq poly(A)-positioning profiling by sequencing aa amino acid AUC area under curve bp base pair CAGE Cap analysis of gene expression CAGE-seq Cap analysis of gene expression sequencing CCDS Consensus CDS CDS Coding sequence CLIP-seq Crosslinking immunoprecipitation sequencing CPAT Coding potential assessment tool CPC Coding potential calculator eCLIP enhanced CLIP FANTOM Functional annotation of the mammalian genome GEO Gene expression omnibus kb kilo base lncRNA long non-coding RNA mESC mouse embryonic stem cell miRNA micro RNA miscRNA miscellaneous RNA MLRF most-likely reading frame mRNA messenger RNA ncRNA non-coding RNA nt nucleotide ORF Open reading frame ROC Receiver operating characteristic RPM Reads per million RPS Ribosome periodicity score rRNA Ribosomal RNA RRS Ribosome release score snoRNA small nucleolar RNA snRNA small nuclear RNA TE Translation efficiency TERIUS Translation-dependent ensemble classifier with ribosome and UPF1 Association score TOC Translated ORF classifier UAS UPF1 association score UniProt Universal protein resource UPF1 Regulator of nonsense transcripts 1 VEGA Vertebrate genome annotation WRE Weighted relative entropy
