# Supplementary material for: TERIUS: accurate prediction of lncRNA via high-throughput sequencing data representing RNA-binding protein association
Source: BMC Bioinformatics. 2018 Feb 19;19(Suppl 1):41. doi: 10.1186/s12859-018-2013-9 (PMC5836835; doi:10.1186/s12859-018-2013-9)
Supplement: Supplementary file 1 — Accession numbers of NCBI GEO data sets used in this study. Table S2. Hyperparameters (kernel, bandwidth, adjustment) of kernel density estimation (PDF 225 kb) [file 12859_2018_2013_MOESM1_ESM.pdf]

Supplementary Table S1. Accession numbers of NCBI GEO data sets used in this study

Species	RPS		UAS		3P-seq	CAGE-seq
	Ribo-seq	RNA-seq	CLIP-seq	RNA-seq		
Human (HeLa)	GSM546920	GSM546921	GSM1163974	GSM1163976	GSM1268942	http://fantom.gsc.riken.jp/5/datafiles/phase1.3/extra/CAGE_peaks/
Mouse (mESC)	-	-	GSM1024301<br>GSM1024302<br>GSM1024303	GSM1024297	GSM1268958	http://fantom.gsc.riken.jp/5/datafiles/phase1.3/extra/CAGE_peaks/

Supplementary Table S2. Hyperparameters (kernel, bandwidth, adjustment) of kernel density estimation

Species	RPS		UAS	
	lncRNA	mRNA	lncRNA	mRNA
Human (HeLa)	cosine, bcv, 1.5	cosine, bcv, 3	epanechnikov, bcv, 1.5	optcosine, nrd0, 2
Mouse (mESC)	-	-	epanechnikov, nrd, 1.5	optcosine, nrd, 1.5
