# Supplementary figures and images for: TERIUS: accurate prediction of lncRNA via high-throughput sequencing data representing RNA-binding protein association
Source: BMC Bioinformatics. 2018 Feb 19;19(Suppl 1):41. doi: 10.1186/s12859-018-2013-9 (PMC5836835; doi:10.1186/s12859-018-2013-9)

a

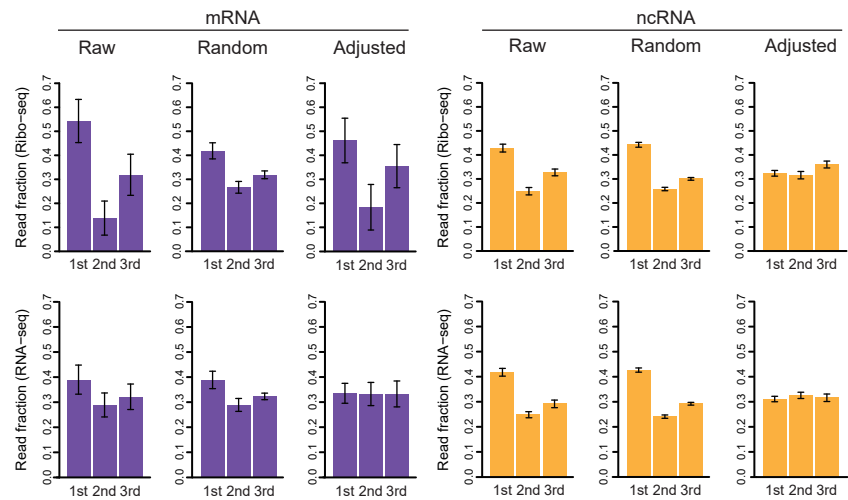

b

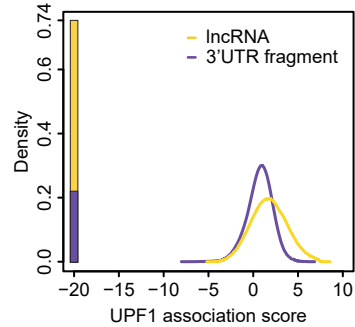

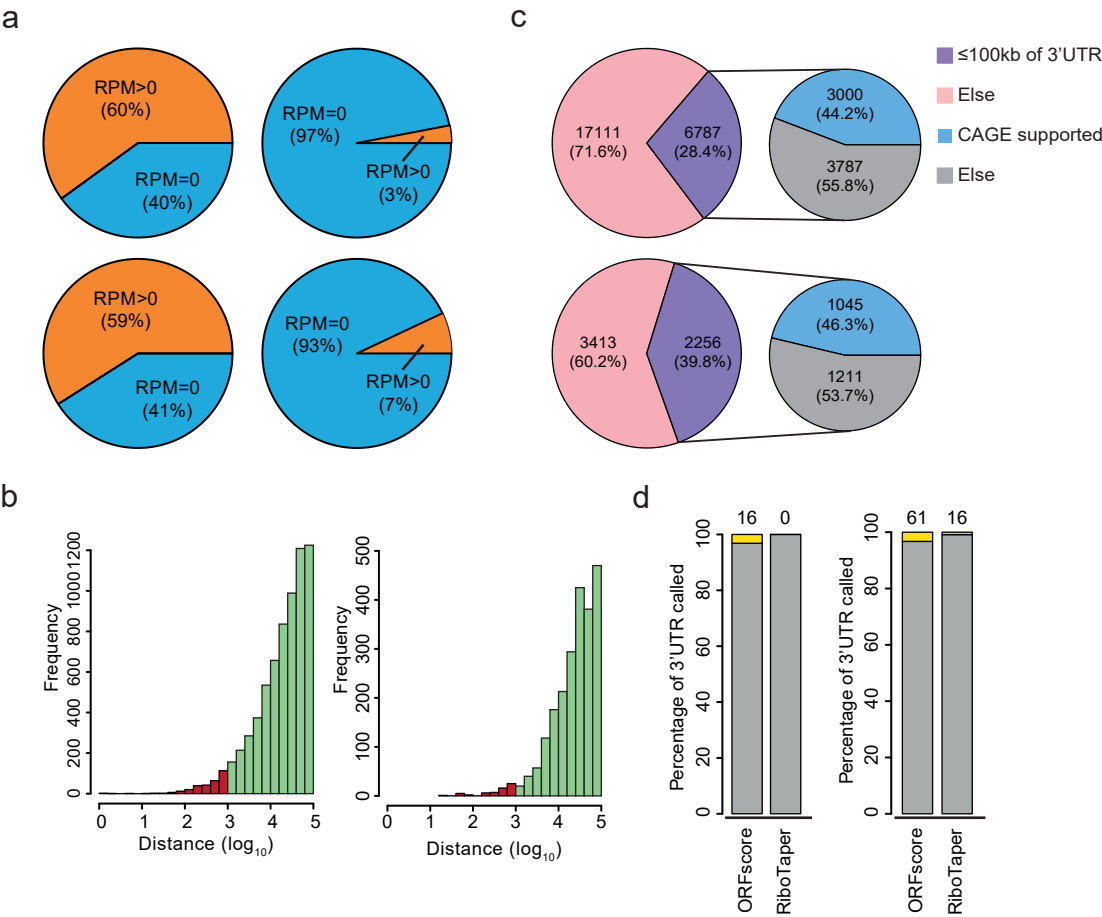

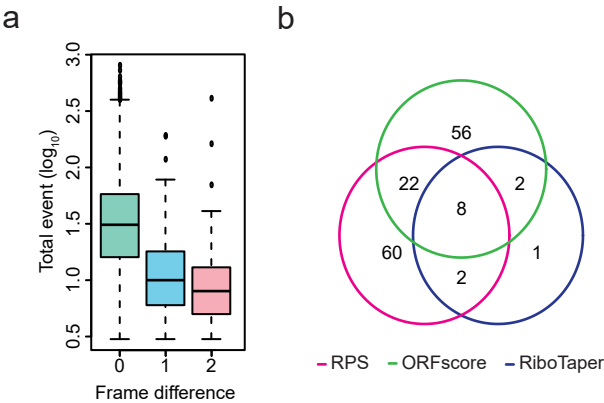

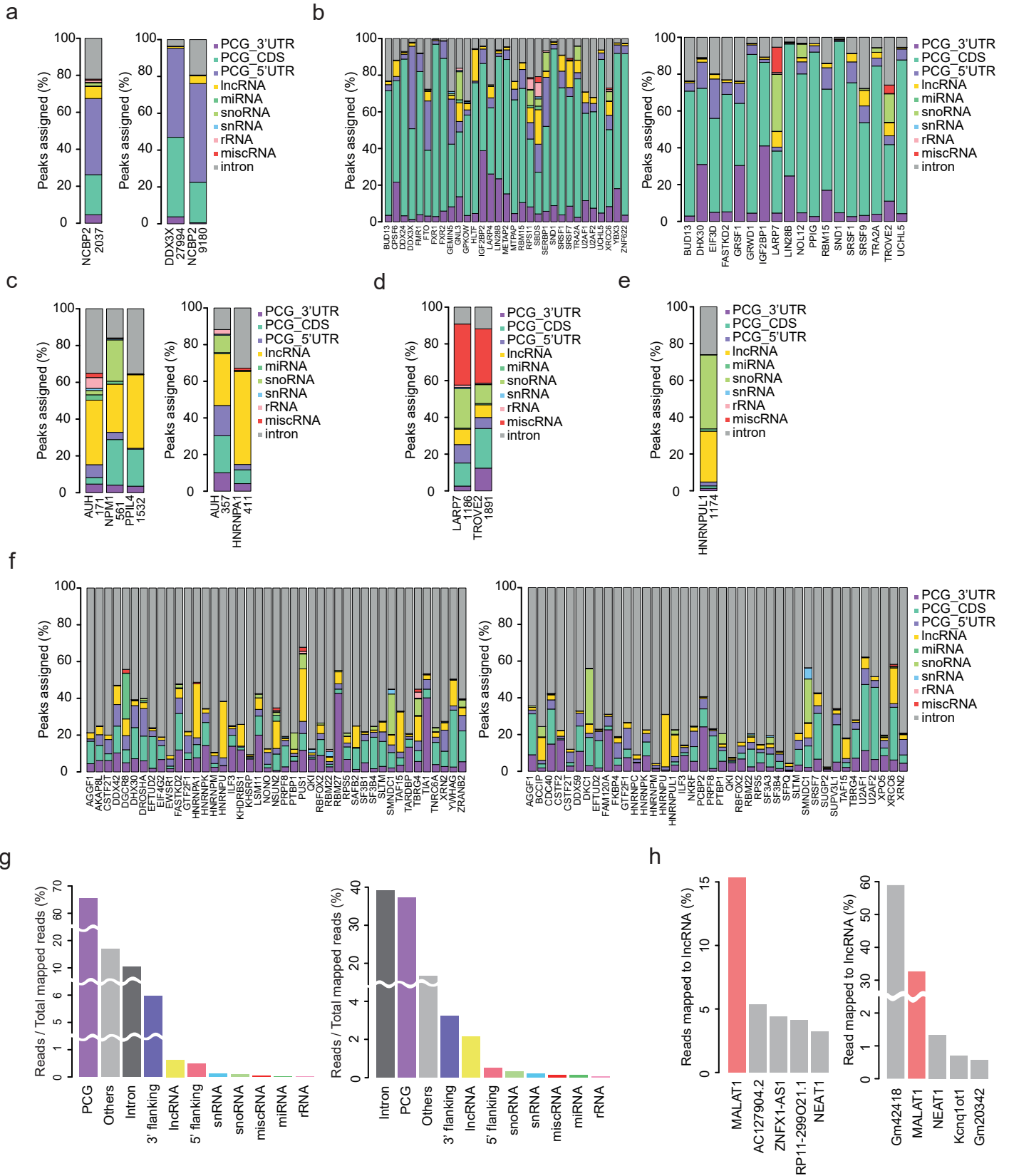

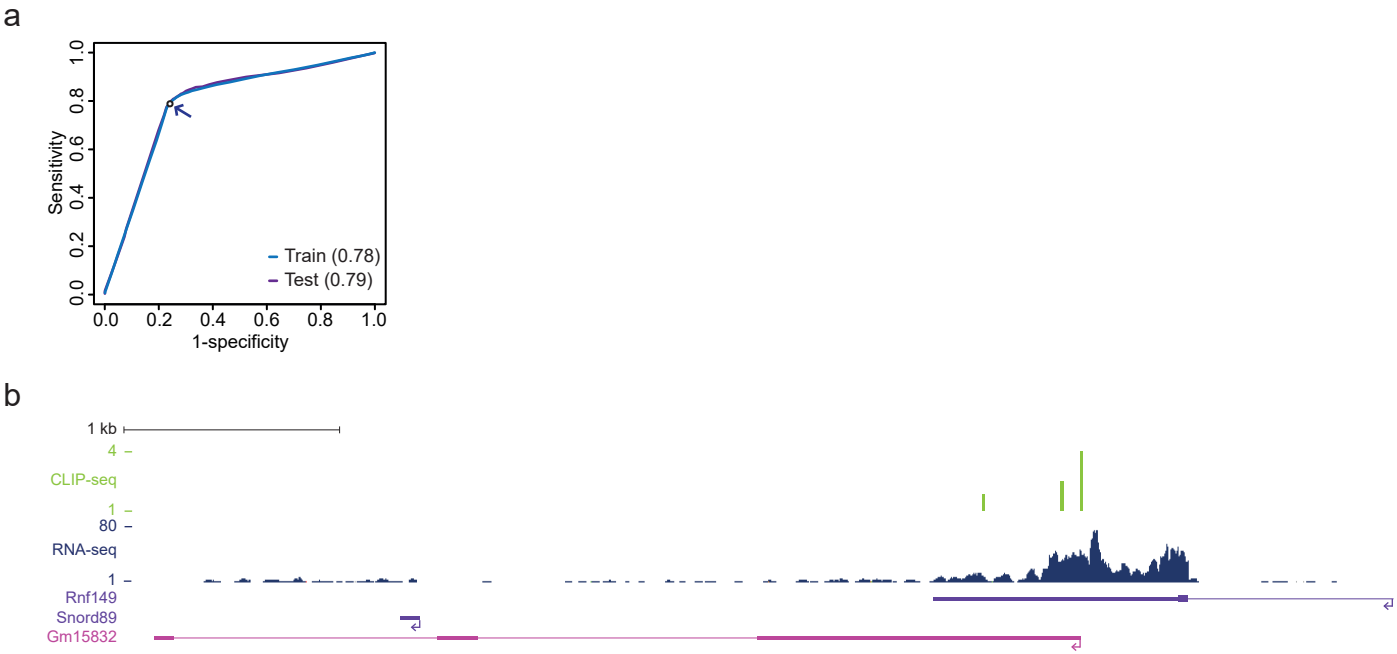

Supplementary Figure S6

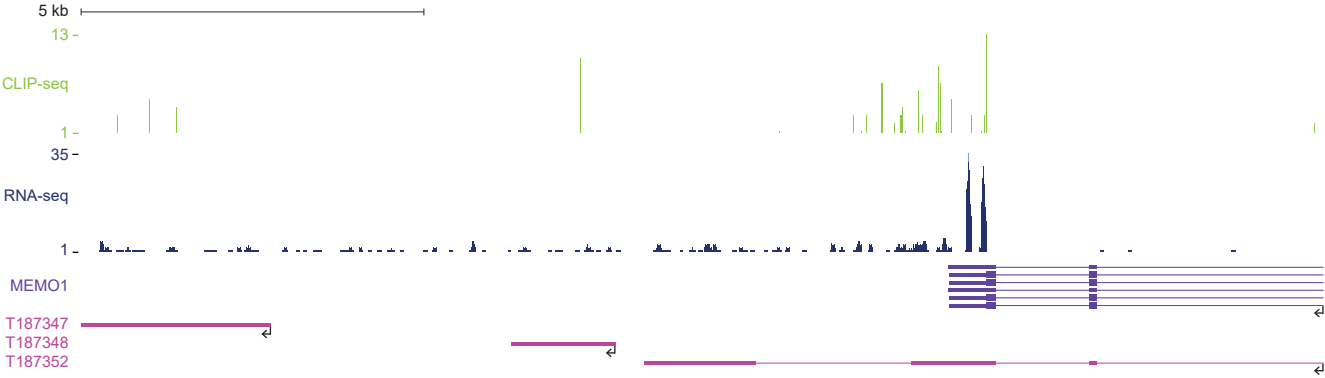

Supplement: Supplementary file 2 — Building RPS and UAS model. (a) Correcting noise in sub-codon position signals. Raw, random and normalized signals of protein-coding genes are colored in purple, and those of ncRNAs are colored in gold. (b) Estimated association density model of lncRNA (gold) and 3’UTR fragments (purple) using mouse data. Figure S2. LncRNAs can be protein-coding or fragments of 3’UTR. (a) Proportion of human protein-coding genes (left) and VEGA lncRNAs (right) associated with Ribo-seq reads (top). Shown in the bottom are same results of mouse genes. (b) Distance between lncRNA start and 3’UTR end of protein-coding genes within 100 kb upstream of lncRNAs (left: human, right: mouse). Frequency of lncRNAs located within 100 kb downstream of 3’UTR are colored in red. (c) Percent of GENCODE v19 lncRNAs (top) located 100 kb downstream of 3’UTR of sense protein-coding gene (purple). Among them, those with CAGE tag supporting their 5’end is shown in blue. Below are the corresponding results of GENCODE vM1 lncRNAs. (d) Classification of 3’UTR regions using ORFscore and RiboTaper. The number on the top of each bars and the portion colored in yellow indicate the number of 3’UTR regions predicted as coding by each method. Figure S3. Performance of RPS compared to ORFscore and RiboTaper. (a) Ribosome read signals of RefSeq protein-coding genes, binned according to the difference between its CDS frame and its predicted frame. (b) Venn diagram depicting GENCODE v19 ncRNA subsets detected by RPS, ORFscore and RiboTaper. Figure S4. Association profiles of RBPs with eCLIP dataset and UPF1 CLIP-seq. Proteins that mostly bind to 5’UTR (a), CDS (b), lncRNA (c), miscRNA (d), snoRNA (e) and intron (f) are shown for eCLIP data of K562 and HepG2 cell lines. snoRNA and miscRNA-dominant proteins are shown for K562 cell lines only. (g) Detailed association profile of UPF1 to various genes in HeLa (left) and mESC (right) cell lines. Flanking 5 and Flanking 3 refers to the region 3 kb outside 5’UTR or 3’UTR. (h) [file 12859_2018_2013_MOESM2_ESM.pdf]
